# The mediation effect of Systemic Immunity Inflammation Index between urinary metals and TOFAT among adults in the NHANES dataset

**DOI:** 10.1038/s41598-024-65925-1

**Published:** 2024-06-28

**Authors:** Weipeng Zhang, Cong Zhang, Dengqiu Lu, Junfeng Nie, Zhumin Hu, Cuiyao Xian, Minxing He

**Affiliations:** 1 The Affiliated Panyu Central Hospital of Guangzhou Medical University, Guangzhou, 511400 Guangdong China; 2 Department of Pharmacy, Guangdong Second Provincial General Hospital, Guangzhou, 510317 Guangdong China

**Keywords:** NHANES, Urinary metal, Total fat, Systemic Immune Inflammatory Index, Mediation, Risk factors, Environmental impact

## Abstract

Systemic Immune Inflammatory Index (SII) is a novel indicator of inflammation. However, no studies have reported the effect of SII on the association between metals and total fat (TOFAT). We aim to investigate the mediated effect of SII on the relationship between urinary metals and TOFAT in a US adult population. This cross-sectional study was conducted among adults with complete information on SII, urine metal concentrations, and TOFAT from the 2011–2018 National Health and Nutrition Examination Survey (NHANES). Multifactorial logistic regression and restricted cubic splines were used to explore the association between urine metal levels and TOFAT. Furthermore, serial mediation analyses were used to investigate the mediating effect of SII on metals and TOFAT. A total of 3324 subjects were included in this study. After adjusting for confounders, arsenic (As), cadmium (Cd), cobalt (Co), cesium (Cs), inorganic mercury (Hg), molybdenum (Mo), manganese (Mn), lead (Pb), antimony (Sb), and thallium(Tl) had negative decreased trends of odds ratios for TOFAT (all *P* for trend < 0.05). In the total population, we found that Cd, Co, and Tu were positively associated with SII (*β* = 29.70, 79.37, and 31.08), whereas As and Hg had a negative association with SII. The mediation analysis showed that SII mediated the association of Co with TOFAT, with the *β* of the mediating effect being 0.9% (95%CI: 0.3%, 1.6%). Our findings suggested that exposure to As, Cd, and Hg would directly decrease the level of TOFAT. However, Co would increase TOFAT, completely mediated by SII, mainly exerted in females rather than males.

## Introduction

With the improvement of living standards, especially in developed countries, such as the United States, obesity has become increasingly common^1^. According to data from the World Health Organization, more and more adults are overweight or obese^2^, with the global prevalence of obesity nearly tripling between 1975 and 2016. Obesity is a metabolic disease that not only causes serious physical health issues but can also increase the incidence of other metabolic diseases such as hypertension and diabetes^3^. This has made obesity a widespread public health problem worldwide. Although the exact causes of obesity are not fully understood, environmental factors may contribute to its development.

Of particular concern is the exposure to harmful metals, which can disrupt human metabolism and affect fat metabolism functions. Metals such as lead (Pb), cadmium (Cd), arsenic (As), manganese (Mn), and barium (Ba) are widely present in the environment, including in the air, soil, and water^4^. People can be exposed to these harmful metals through various means, such as consuming contaminated food, drinking contaminated water, and inhaling polluted air. These metals are associated with adverse health effects, including sarcopenia, osteoporosis, kidney damage, etc.^5–7^. Previous studies have reported that exposure to As, Cd, cobalt (Co), Pb, and mercury (Hg) could cause chronic diseases such as diabetes and obesity^8–10^.

Inflammation is the body's response to harmful foreign substances and results from the interaction between the organism and the environment^11^. Systemic inflammation can be directly measured through blood biochemical markers or derived ratios^12^. Discovering novel inflammatory indices is crucial for studying various diseases. Many studies have indicated that the Systemic Immunity Inflammation Index (SII) can assess tissue and cell damage caused by injury factors. SII is calculated by multiplying the platelet count by the neutrophil count and dividing by the lymphocyte count^13^. Research has shown that SII is associated with many chronic diseases. Mahemuti et al.^12^ found a remarkable correlation between SII and hyperlipidemia. Xu et al.^14^ showed that SII influenced mortality in cardiovascular disease patients. Chen et al.^15^ also demonstrated that combining SII and other clinical data could predict the risk of death in senior cardiovascular patients.

Changes in inflammatory indicators may result from the toxic effects of hazardous metals^16^. For example, Cd exposure can activate inflammatory pathways and cause chronic inflammation^17^. Epidemiological studies by Zhong et al.^18^ have shown that SII is associated with exposure to Mn, Cd, and Pb. Additionally, obesity is linked to fat inflammation^19^, where immune cell infiltration in visceral fat contributes to adverse metabolic complications^20^. Some studies have revealed that obesity is associated with altered white blood cell counts^21^.

Building on these studies, we hypothesized that SII might play a role in the relationship between metals and total body fat (TOFAT). However, the specific role of SII in this context has not been reported. Therefore, using the data from the National Health and Nutrition Examination Survey (NHANES), we first investigated the association between metal exposure and TOFAT. We then further examined the mediating effect of SII on the relationship between metals and TOFAT.

## Methods

### Study population

**Figure 1:**
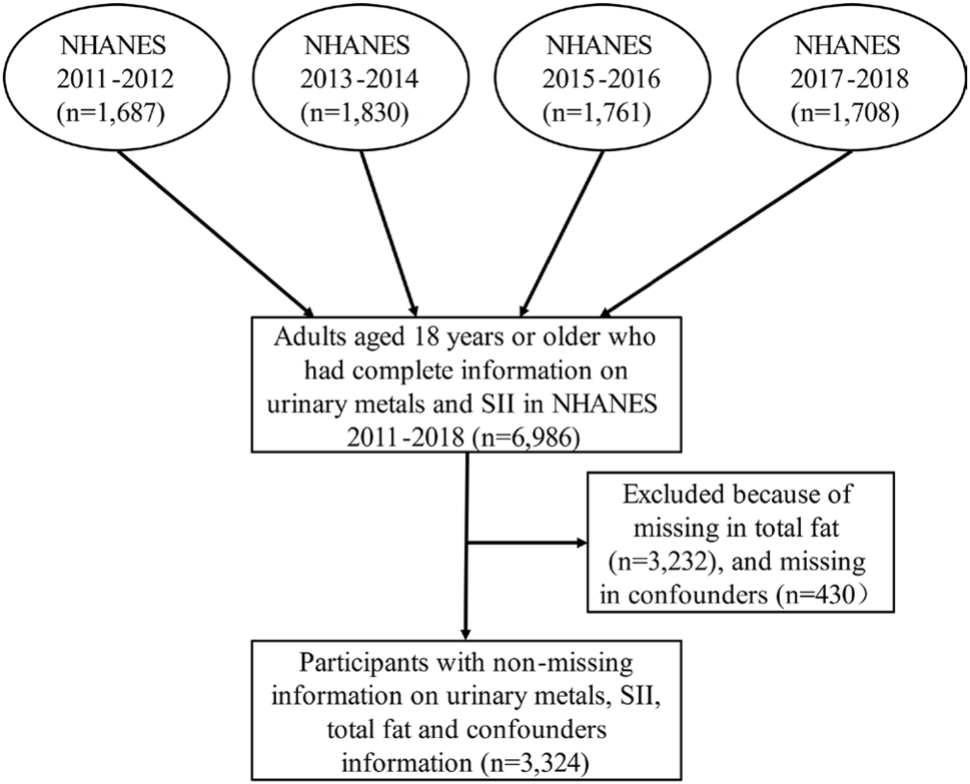
Schematic diagram of study methodology in NHANES 2011 2018.

The NHANES is a multistage stratified composite design survey, which started in the 1980s, and the biological samples collected in this study contain serum, plasma, and urine from participants, covering a wide range of measures. The study also contains a large amount of data from questionnaires covering demographic, dietary, and health-related issues, and a physical examination section that includes a variety of laboratory testing data, radiologic testing data, etc., to assess the health status of people in the United States^22^. The NHANES collects data on about 5000 persons each year^23^. We combined 4 consecutive NHANES surveys, 2011–2012, 2013–2014 2015–2016, and 2017–2018, into a single analytic sample. A total of 6,986 subjects aged 18 years or older and with complete information on urinary metals and SII were included. There were 3232 participants without TOFAT excluded in this study. Then, the participants who had no data on education level, body mass index (BMI), alcohol use, smoking behavior, physical activity, sedentary and nutrient intakes (n = 430) were also excluded, and the final study population of this study was 3,324. The process of recruiting was shown in Fig. 1.

### Urinary metal detections

13 metals were detected in urine [As (total arsenic), Ba, Cd, Co, cesium(Cs), Hg (inorganic), molybdenum(Mo), Mn, Pb, antimony(Sb), tin(Sn), thallium(Tl), and tungsten(Tu)]. All metals were analyzed by inductively coupled plasma dynamic reaction cell mass spectrometry, Laboratory Sciences Division, National Center for Environmental Health, Atlanta, Georgia. Metal concentrations below the limit of detection were calculated by dividing the limit of detection by the root sign 2^24^. We used these estimates in our analyses, consistent with the Centers for Disease Control and Prevention’s National Report on Human Exposure to Environmental Chemicals. All urinary metals were corrected for urinary creatinine (Cr) and reported as μg/g Cr^25^.

### TOFAT and SII assessment

TOFAT was measured by dual-energy x-ray absorptiometry (DXA), a commonly utilized method of measuring body composition because of its rapid detection, simplicity of operation, and low radiation exposure. The use of DXA allowed for the detection of data on bone density and soft tissue counts throughout the body. In NHANES, DXA was used to measure the total fat of the body in participants between the ages of 8–59. Based on previous research, the final analysis included possible confounding factors associated with TOFAT^26^. Blood samples were transported to the NHANES Mobile Examination Centers for final determination. Detailed descriptions of the sample collection, transportation, and processing were presented in the NHANES Laboratory/Medical Technician Procedures Manual. Automated blood analysis equipment was used to measure lymphocyte, neutrophil, and platelet counts, and finally, the SII level was calculated by multiplying the platelet count by the neutrophil count/lymphocyte count^12^.

### Covariates assessment

According to a statement on the NHANES website, demographic and lifestyle information about the population was collected by specialized personnel. The covariates we included age, gender, BMI, race, education level, drinking, smoking, physical activity, sedentary, and nutrient intake. Age was divided into < 45 years and ≥ 45 years. BMI was divided into normal (18.5–24.9 kg/m^2^) and abnormal (< 18.5 kg/m^2^ or ≥ 25.0 kg/m^2^). The race was divided into Hispanic, non-Hispanic-White, non-Hispanic-Blac, and non-Hispanic other. Education level divided into was less than high school degree, high school degree, and more than high school degree. Alcohol use was divided into never drinker, ever drinker, and current drinker. Smoking was divided into never smoker, ever smoker, and current smoker^27^. Daily exercise was divided into < 100 min MVPA (moderate to vigorous physical activity) and ≥ 100 min MVPA^28^. Sedentary was divided into < 360 min and ≥ 360 min. In addition, according to the recommended range of energy intake^29^, energy was divided into < 2050 kcal, 2050–3050 kcal, and ≥ 3051 kcal. The ratios of carbohydrates, fats, and proteins for supplying energy to the human body are as follows: carbohydrates should account for 55–60% of the total energy, fats should account for 25–30% of the total energy, and proteins should account for 10–15% of the total energy, and so we stratify the three substances according to the ratios. In addition, the results of the current study found a co-linearity between BMI and TOFAT (VIF = 6.370) so BMI was excluded for adjustment^30^.

### Statistical analysis

We followed NHANES guidelines and accounted for complex survey design factors, including sample weights, clustering, and stratification. All statistical analyses were conducted by standards from the Centers for Disease Control and Prevention. Categorical parameters were reported as proportions, and continuous variables were expressed as inter-quartile range (IQR). Demographic characteristics of subjects were evaluated using chi-square, rank sum tests, and correlation analysis. Nonparametric tests were used to compare differences in metal concentrations between groups. Multivariable logistic regression was conducted to explore the associations of metals and SII with TOFAT, obtaining odds ratios (OR) and corresponding 95% confidence interval (CI). Trend tests were conducted to assess the relationship between metals and TOFAT. The restricted cubic splines (RCS) were used to explore the dose–response relationship between metals and TOFAT. Multivariable linear regression was used to explore the associations of metals with SII. We also used g-computation analysis to explore the mixture effect of metals on TOFAT and SII. Finally, the criteria under which mediation analysis can be performed are: “statistically significant correlation between X and M” and “statistically significant correlation between M and Y”^31^. Once the conditions for mediation analysis were met, we used the R language package “bruceR” to analyze the mediating role of SII in the relationship between metals and TOFAT. The mediation analysis was carried out using the Bayesian Monte Carlo method to avoid the writing of fitting algorithms by specifying the observation and state equations for each model^32^. The number of simulations in this study was set to 1000 and the seed was set to 1, to repeat the analysis to obtain consistent results. The direct effect meant that the metal had a direct effect on TOFAT and the indirect effect meant that the metal affected TOFAT through the mediator^33^. Directed acyclic graphs were used to confirm the confounding variables adjusted in the analysis, and the results were shown in Fig. S1. A two-sided value of *P* of < 0.05 was regarded as statistically significant. All statistical analyses were performed with SPSS (version 24.0) or R (version 4.3.1).

### Ethics statement

The study was approved by the National Center for Health Statistics Research Ethics Review Board. The study was conducted in accordance with the local legislation and institutional requirements. The participants provided their written informed consent to participate in this study.

## Results

### Characteristics of participants and metals distribution

We included 3324 adults aged 18 years or older in our research. The demographic characteristics of the study subjects with low TOFAT or high TOFAT were listed in Table 1. Overall, gender, BMI, race, smoking, physical activity, energy intake, carbohydrate intake, and protein intake were statistically different between low TOFAT and high TOFAT participants. Participants with high TOFA were more likely to be female than those with low TOFAT. These subjects were also more likely to have an abnormal BMI and less time for physical activity. A difference has existed in SII between the low TOFAT group and the high TOFAT group (Table 1). As shown in Table S1, we also reported the demographic information of males and females. BMI, smoking, physical activity, and the intake of fat were different across TOFAT status in males and females. The geometric mean (GM) and quartiles of metal concentrations were shown in Table 2. The concentrations of Cd, Co, and Sn were higher in the high TOFAT group than in the low TOFAT group [GM (μg/g Cr): 0.166 vs 0.155, 0.388 vs 0.369, and 0.483 vs 0.400], while the opposite was observed for As and Pb [GM (μg/g Cr): 0.064 vs 0.079 and 0.288 vs 0.317]. The correlation analysis between the log-transform level of urinary metals was reported in Fig. S2.

**Table 1:** Characteristics of study population in NHANES 2011–2018 (n = 3,324).

Baseline characteristics	Total (n = 3324)	TOFAT		*P*
		Low (n = 1662)	High (n = 1662)	
Age (%)				0.855
< 45 year	2194 (66.0)	1100 (66.2)	1094 (65.8)	
≥ 45 year	1130 (34.0)	562 (33.8)	568 (34.2)	
Gender (%)				< 0.001
Male	1676 (50.4)	926 (55.7)	750 (45.1)	
Female	1648 (49.6)	736 (44.3)	912 (54.9)	
BMl (%)				< 0.001
Normal	1012 (30.4)	560 (33.7)	452 (27.2)	
Abnormal	2312 (69.6)	1102 (66.3)	1210 (72.8)	
Race (%)				< 0.001
Mexican American	546 (16.5)	249 (15.0)	297 (17.9)	
Other Hispanic	349 (10.5)	149 (9.0)	200 (12.0)	
Non-Hispanic White	1184 (35.6)	526 (31.6)	658 (39.6)	
Non-Hispanic Black	665 (20.0)	431 (25.9)	234 (14.1)	
Non-Hispanic other	580 (17.4)	307 (18.5)	273 (16.4)	
Education level (%)				0.660
Less than high school degree	613 (18.4)	298 (17.9)	315 (19.0)	
High school degree	775 (23.3)	384 (23.1)	391 (23.5)	
More than high school degree	1936 (58.2)	980 (59.0)	956 (57.5)	
Drinking status (%)				0.533
Never drinker	480 (14.4)	242 (14.6)	238 (14.3)	
Ever drinker	356 (10.8)	1252 (75.3)	1236 (74.4)	
Current drinker	2488 (74.8)	168 (10.1)	188 (11.3)	
Smoking status (%)				0.018
Never smoker	2070 (62.3)	1056 (63.5)	1014 (61.0)	
Ever smoker	542 (16.3)	323 (19.4)	389 (23.4)	
Current smoker	712 (21.4)	283 (17.0)	259 (15.6)	
Physical activity (%)				0.002
< 100 min MVPA	2312 (69.6)	1115 (67.1)	1197 (72.0)	
≥ 100 min MVPA	1012 (30.4)	547 (32.9)	465 (28.0)	
Sedentary (%)				0.297
< 360 min	1583 (47.6)	807 (48.6)	776 (46.7)	
≥ 360 min	1741 (52.4)	855 (51.4)	886 (53.3)	
Nutrient Intakes				
Energy (%)				0.001
< 2050 kcal	1635 (49.2)	764 (46.0)	871 (52.5)	
2050–3050 kcal	1088 (32.7)	587 (35.3)	501 (30.1)	
≥ 3051 kcal	601 (18.1)	311 (18.7)	290 (17.4)	
Protein (%)				0.019
< 51.5 gm	719 (21.6)	333 (20.0)	386 (23.2)	
51.5–91.5 gm	1356 (40.8)	670 (40.3)	686 (41.3)	
≥ 91.6 gm	1249 (37.6)	659 (39.7)	590 (35.5)	
Carbohydrate (%)				0.001
< 282.0 gm	2117 (63.7)	1009 (60.7)	1108 (66.7)	
282.0–457.5 gm	932 (28.0)	511 (30.7)	421 (25.3)	
≥ 457.6 gm	275 (8.3)	142 (8.6)	133 (8.0)	
Fat (%)				0.061
< 45.5 gm	615 (18.5)	281 (16.9)	334 (20.1)	
45.5–101.5 gm	1683 (50.6)	858 (51.6)	825 (49.6)	
≥ 101.6 gm	1026 (30.9)	523 (31.5)	503 (30.3)	
SII (10^3^/μL)	444 (312, 617)	312 (249, 375)	617 (517, 765)	< 0.001

**Table 2:** Geometric means and quartile of urinary metal concentrations in NHANES (n = 3324).

Urinary metals (μg/g Cr)	Low TOFAT					High TOFAT					*P*
	GM	Q1	Q2	Q3	Q4	GM	Q1	Q2	Q3	Q4	
As	0.079	< 0.038	0.038–0.065	0.065–0.141	> 0.141	0.064	< 0.033	0.033–0.053	0.053–0.105	> 0.105	< 0.001
Ba	1.072	< 0.625	0.625–1.093	1.093–1.870	> 1.870	1.110	< 0.593	0.593–1.165	1.165–2.095	> 2.095	0.131
Cd	0.155	< 0.081	0.081–0.143	0.143–0.296	> 0.296	0.166	< 0.089	0.089–0.163	0.163–0.297	> 0.297	0.013
Co	0.369	< 0.242	0.242–0.350	0.350–0.522	> 0.522	0.388	< 0.242	0.242–0.3785	0.379–0.585	> 0.585	0.011
Cs	4.056	< 2.859	2.859–3.9566	3.957–5.7013	> 5.701	4.008	< 2.941	2.941–3.900	3.900–5.371	> 5.371	0.440
Hg	0.003	< 0.001	0.001–0.003	0.003–0.005	> 0.005	0.002	< 0.001	0.001–0.002	0.002–0.005	> 0.005	0.008
Mo	35.509	< 24.095	24.095–35.750	35.750–52.706	> 52.706	34.297	< 23.300	23.300–34.454	34.454–50.190	> 50.190	0.066
Mn	0.126	< 0.072	0.072–0.116	0.116–0.200	> 0.200	0.119	< 0.067	0.067–0.110	0.110–0.1972	> 0.197	0.087
Pb	0.317	< 0.188	0.188–0.305	0.305–0.516	> 0.516	0.288	< 0.175	0.175–0.281	0.281–0.448	> 0.448	< 0.001
Sb	0.051	< 0.031	0.031–0.047	0.047–0.073	> 0.072	0.050	< 0.031	0.031–0.046	0.046–0.071	> 0.071	0.521
Sn	0.400	< 0.208	0.208–0.368	0.368–0.693	> 0.693	0.483	< 0.244	0.244–0.427	0.427–0.859	> 0.859	< 0.001
Tl	0.162	< 0.110	0.110–0.158	0.158–0.228	> 0.228	0.159	< 0.113	0.113–0.157	0.157–0.224	> 0.224	0.899
Tu	0.064	< 0.036	0.036–0.061	0.061–0.106	> 0.106	0.063	< 0.035	0.035–0.059	0.059–0.105	> 0.105	0.449

GM, geometric mean.

### Associations between metal concentrations and TOFAT

**Figure 2:**
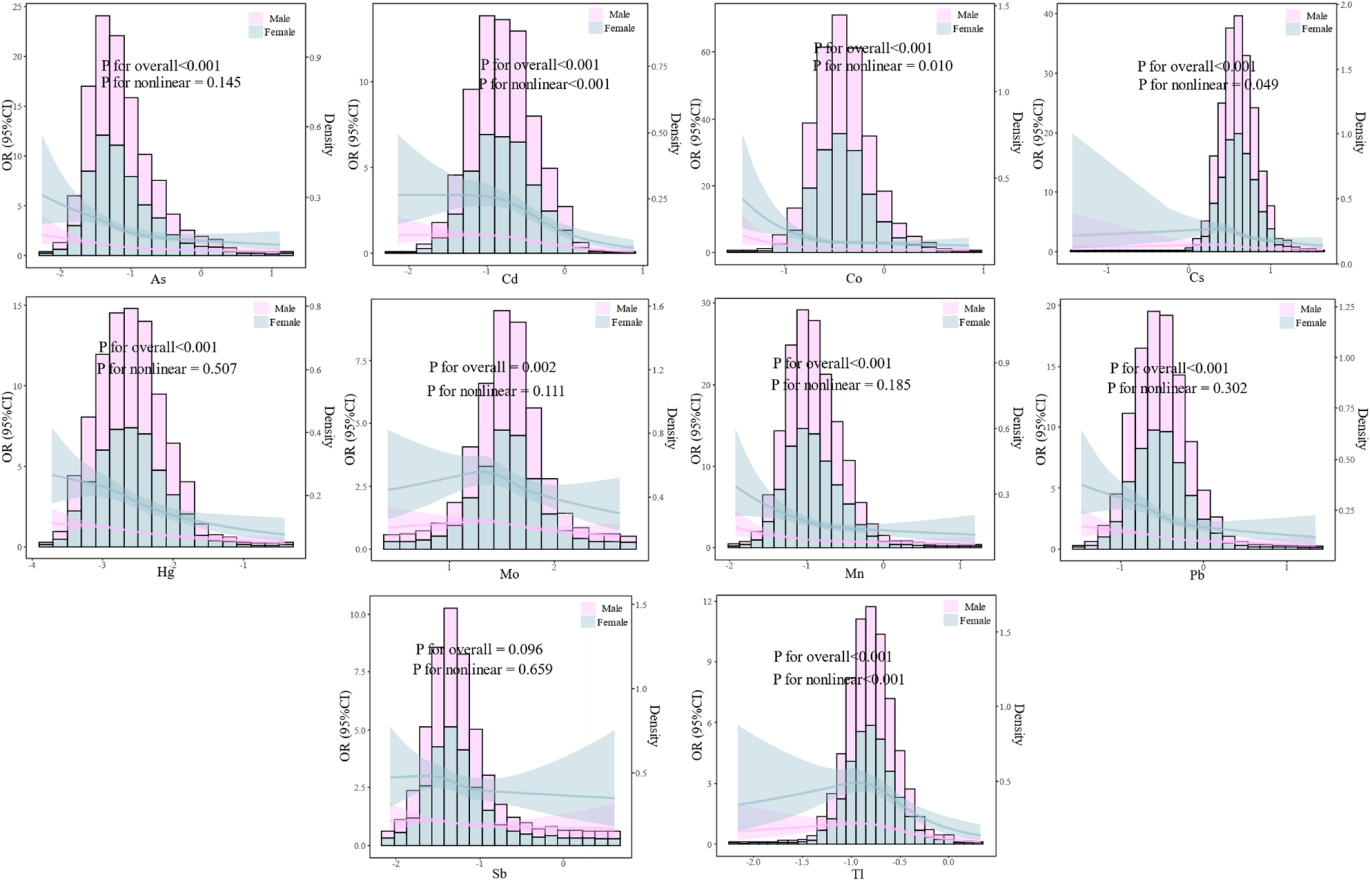
Adjusted restricted cubic spline (RCS) for the association between the log transformed level of urinary metals and TOFAT.

The associations between the level of metals and TOFAT were shown in Tables 3 and S2. As shown in model 3, after adjusting for confounders, 10 metals (As, Cd, Co, Cs, Hg, Mo, Mn, Pb, Sb, and Tl) had negative decreased trends of OR for TOFAT (all *P* for trend < 0.05). Compared with participants in the lowest quartile of As, Cd, Co, Cs, Hg, Mo, Mn, Pb, Sb, and Tl, participants in the highest quartile showed 59.2% (95%CI: 0.329, 0.505), 55.7% (95%CI: 0.342, 0.574), 31.8% (95%CI: 0.546, 0.852), 49.5% (95%CI: 0.407, 0.627), 50.9% (95%CI: 0.396, 0.608), 27.9% (95%CI: 0.586, 0.887), 46.0% (95%CI: 0.437, 0.667), 58.4% (95%CI: 0.330, 0.524) 19.1% (95%CI: 0.661, 0.989), and 27.3% (95%CI: 0.587, 0.900) decreased of TOFAT, respectively. In addition, RCS analyses showed nonlinear associations between Cd, Co, Cs, Tl, and TOFAT (with *P*-values for the nonlinearity of < 0.05). Non-linear associations were consistent between males and females at different metal concentrations (Fig. 2). The mixture effect of metals on TOFAT was shown in Fig. S3(a), and we found that Pb, Sn, As, Cd, and Co had a greater effect on TOFAT.

**Table 3:** The logistic regression of metals and TOFAT in NHANES.

Metals	Model	OR (95%CI)				*P* for trend
		Q1	Q2	Q3	Q4	
As	Model 1	Ref.	0.749 (0.623, 0.900)	0.733 (0.607, 0.884)	0.531 (0.435, 0.650)	< 0.001
	Model 2	Ref.	0.684 (0.565, 0.830)	0.643 (0.528, 0.783)	0.417 (0.337, 0.515)	< 0.001
	Model 3	Ref.	0.680 (0.561, 0.825)	0.633 (0.519, 0.772)	0.408 (0.329, 0.505)	< 0.001
Cd	Model 1	Ref.	1.245 (1.046, 1.483)	1.430 (1.187, 1.723)	1.089 (0.887, 1.338)	0.043
	Model 2	Ref.	0.914 (0.759, 1.102)	0.839 (0.682, 1.032)	0.465 (0.363 0.596)	< 0.001
	Model 3	Ref.	0.903 (0.749, 1.089)	0.811 (0.656, 1.002)	0.443 (0.342, 0.574)	< 0.001
Co	Model 1	Ref.	0.846 (0.700, 1.022)	0.989 (0.818, 1.195)	1.304 (1.074, 1.583)	0.004
	Model 2	Ref.	0.707 (0.580, 0.863)	0.692 (0.565, 0.849)	0.684 (0.548, 0.853)	0.001
	Model 3	Ref.	0.709 (0.581, 0.865)	0.693 (0.565, 0.849)	0.682 (0.546, 0.852)	0.001
Cs	Model 1	Ref.	1.171 (0.973, 1.409)	1.153 (0.954, 1.392)	0.887 (0.731, 1.078)	0.336
	Model 2	Ref.	0.998 (0.821, 1.211)	0.800 (0.654, 0.980)	0.515 (0.415, 0.638)	< 0.001
	Model 3	Ref.	0.991 (0.816, 1.204)	0.789 (0.644, 0.967)	0.505 (0.407, 0.627)	< 0.001
Hg	Model 1	Ref.	0.976 (0.808, 1.179)	0.888 (0.734, 1.074)	0.778 (0.640, 0.947)	0.008
	Model 2	Ref.	0.824 (0.676, 1.005)	0.664 (0.542, 0.813)	0.501 (0.405, 0.620)	< 0.001
	Model 3	Ref.	0.818 (0.671, 0.998)	0.654 (0.534, 0.802)	0.491 (0.396, 0.608)	< 0.001
Mo	Model 1	Ref.	0.989 (0.821, 1.192)	0.925 (0.765, 1.117)	0.859 (0.706, 1.045)	0.102
	Model 2	Ref.	0.981 (0.808, 1.190)	0.863 (0.708, 1.050)	0.731 (0.595, 0.898)	0.002
	Model 3	Ref.	0.974 (0.802, 1.182)	0.856 (0.702, 1.043)	0.721 (0.586, 0.887)	0.001
Mn	Model 1	Ref.	0.820 (0.678, 0.993)	0.799 (0.659, 0.967)	0.855 (0.704, 1.038)	0.103
	Model 2	Ref.	0.663 (0.543, 0.811)	0.580 (0.472, 0.711)	0.541 (0.438, 0.668)	< 0.001
	Model 3	Ref.	0.662 (0.542, 0.810)	0.575 (0.469, 0.706)	0.540 (0.437, 0.667)	< 0.001
Pb	Model 1	Ref.	0.873 (0.732, 1.040)	0.864 (0.715, 1.044)	0.632 (0.515, 0.775)	< 0.001
	Model 2	Ref.	0.731 (0.607, 0.880)	0.624 (0.508, 0.766)	0.421 (0.336, 0.529)	< 0.001
	Model 3	Ref.	0.725 (0.601, 0.874)	0.615 (0.499, 0.757)	0.416 (0.330, 0.524)	< 0.001
Sb	Model 1	Ref.	1.076 (0.888, 1.303)	1.015 (0.838, 1.230)	0.939 (0.775, 1.138)	0.435
	Model 2	Ref.	0.972 (0.797, 1.187)	0.869 (0.711, 1.061)	0.801 (0.656, 0.980)	0.017
	Model 3	Ref.	0.975 (0.799, 1.190)	0.873 (0.714, 1.067)	0.809 (0.661, 0.989)	0.022
Tl	Model 1	Ref.	1.230 (1.012, 1.494)	1.121 (0.921, 1.364)	1.073 (0.879, 1.308)	0.793
	Model 2	Ref.	1.160 (0.947, 1.421)	0.869 (0.707, 1.069)	0.741 (0.599, 0.917)	< 0.001
	Model 3	Ref.	1.154 (0.942, 1.414)	0.857 (0.696, 1.055)	0.727 (0.587, 0.900)	< 0.001

Model 1: no covariates were adjusted; Model 2: age and gender were adjusted; Model 3: age, gender, smoking, and drinking were adjusted.

### Associations between metal concentrations and SII

**Figure 3:**
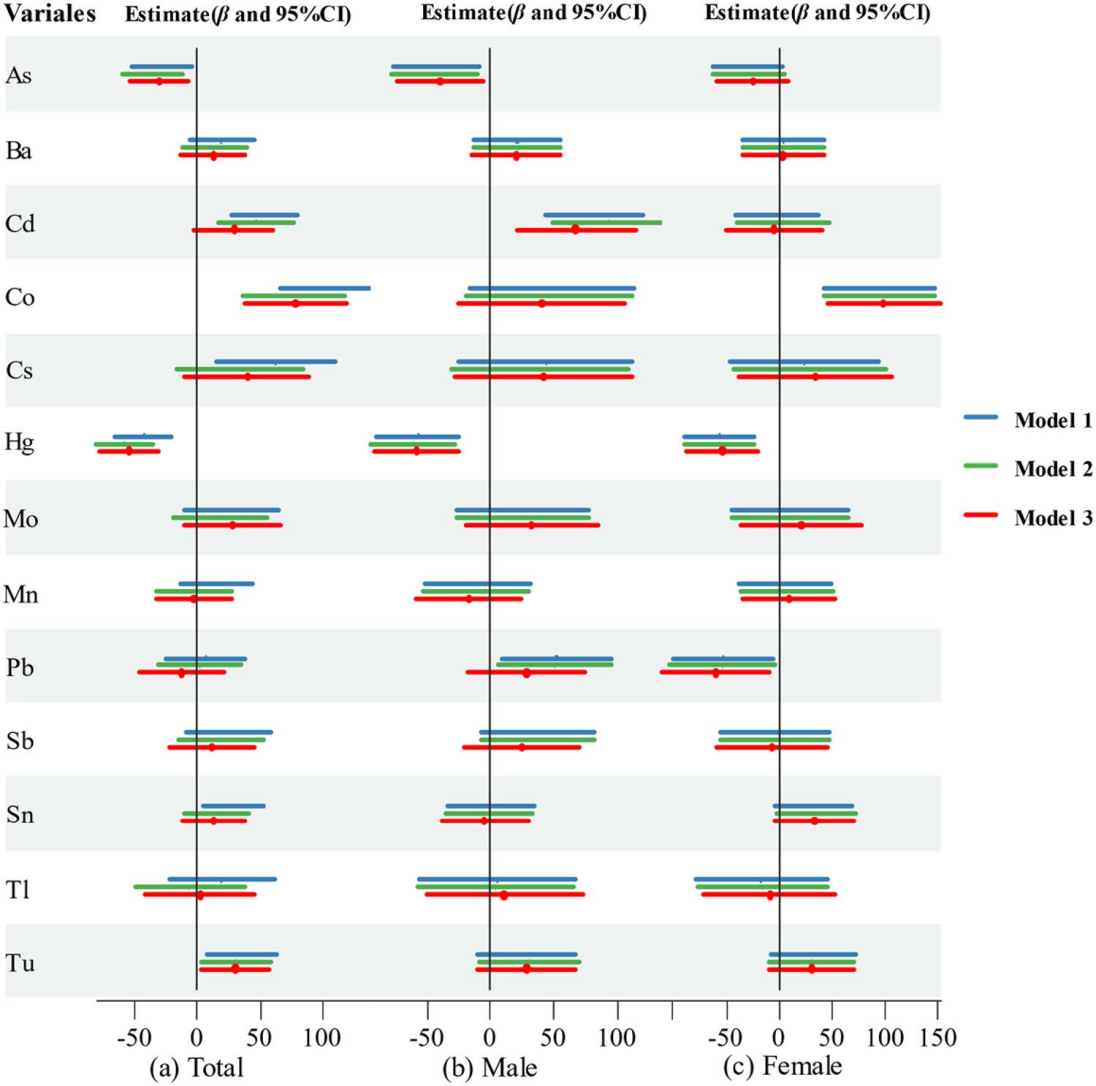
Linear regression results between log transformed metal concentration and SII.

Figure 3 showed the relationship between metal concentrations and SII in the total, male, and female populations. In the total population, We found a positive connection between Cd, Co, Tu, and SII in the no covariate-adjusted models (model 1)[*β* = 54.48 (95%CI: 28.12, 80.84); *β* = 104.33 (95%CI: 67.59, 141.06); *β* = 35.90 (95%CI: 8.20, 63.60)] and in the fully adjusted model (model 3) [*β* = 29.70 (95%CI: − 2.46.65, 61.86); *β* = 79.37 (95%CI: 38.64, 120.11); *β* = 31.08 (95%CI: 3.36, 58.79)]. We also found that the reduction of SII was 35.03-fold (95%CI: − 58.81, − 11.25) and 58.12-fold (95%CI: − 81.41, − 34.83) for each unit increase in As and Hg concentrations. In males, the metal with which we found a statistically positive relationship with SII was only Cd [*β* = 68.01 (95%CI: 21.16, 114.86)], while in females, that was Co [*β* = 99.37 (95%CI: 46.47, 152.28)]. In males, the metals negatively associated with SII were As and Hg, while in females they were Hg and Pb. The mixture effect of metals on SII was shown in Fig. S3(b), we found that Hg, Cd, Co, Pb, and Tu contributed more to SII.

### Associations between SII and TOFAT

Table S3 showed the associations between SII and TOFAT based on the multivariate logistic regression model. In the total, male, and female populations, SII always had a positive trend with TOFAT (*P* for trend < 0.001). In the total population, subjects in the highest quartile had an 82.1% increase in TOFAT compared to subjects in the lowest quartile of SII (Model 3), whereas TOFAT increased by 50.8% and 124.6% for males and females, respectively.

### Mediation analyses

**Figure 4:**
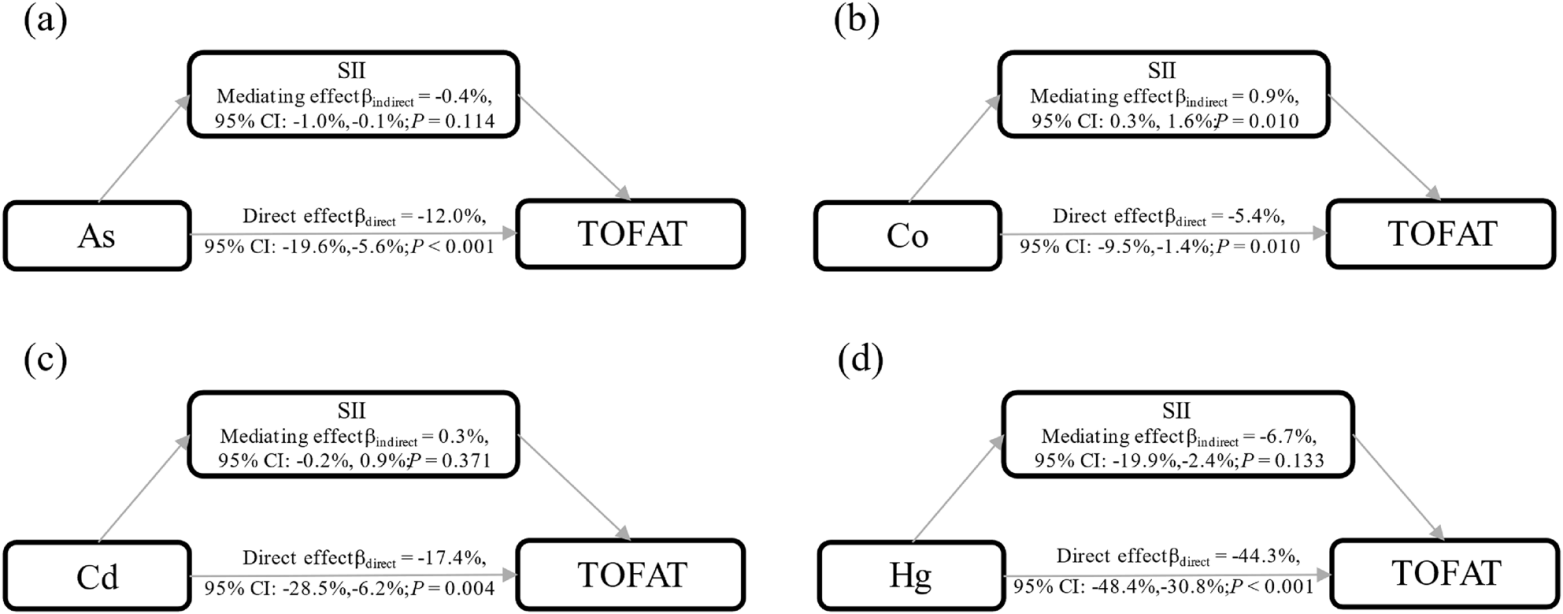
Estimated proportion of the association between As, Co, Cd, Hg, and TOFAT mediated by SII in the total participants. Models were adjusted for age, gender, smoking, drinking, physical activity, sedentary, and nutrient intake.

Based on the above results, the mediation analyses were performed. In the total population, after adjusting for confounders, SII had a mediated effect on the associations of Co with TOFAT, and the *β* of mediating effect was 0.9% (95%CI: 0.3%, 1.6%). The mediating effects of SII were not found on the associations of As, Cd, and Hg with TOFAT, with the *β* of mediating effect were − 0.4% (95%CI: − 1.0%, − 0.1%), 0.3% (95%CI: − 0.2%, 0.9%), and − 6.7% (95%CI: − 19.9%, 2.4%), respectively. Moreover, the direct effect coefficients of the associations between As, Co, Cd, Hg, and TOFAT were − 12.0% (95%CI: − 19.6%, − 5.6%), − 5.4% (95%CI: − 9.5%, − 1.4%), − 17.4% (95%CI: − 28.5%, − 6.2%), and − 44.3% (95%CI: − 48.4%, − 30.8%), respectively (Fig. 4). In males, SII was found to have a mediated role in the relationship between Cd, Hg, and TOFAT [*β*_indirect_ = 1.3% (95%CI: 0.3%, 2.9%) and *β*_indirect_ = − 13.9% (95%CI: − 20.1%, − 2.4%)] (Fig. S4). In females, SII was found to have a mediated effect on the associations of Co with TOFAT [*β*_indirect_ = 1.3% (95%CI: 0.5%, 2.3%)] (Fig. S5).

## Discussion

To the best of our knowledge, no current reports have explored the role of SII in the relationship between metals and TOFAT. Our study employed statistical methods, including linear and logistic regression, to investigate the relationship between metals, SII, and TOFAT, and to determine whether the conditions for mediation analysis were met. We then used mediation analysis with the quasi-Bayesian Monte Carlo method to examine the associations among metals, SII, and TOFAT in the total, male, and female populations. The results of our mediation analysis were satisfactory, providing valuable insights into the relationships.

Although the underlying mechanisms are not fully understood, it is plausible that metals play an important role in the metabolism of TOFAT. Interestingly, our study found that most metals had a negative trend with TOFAT, consistent with results from several other studies. For example, Vrijheid et al. found that Co was negatively associated with BMI in a study on environmental pollutants and childhood obesity^34^. Similarly, Su et al.^35^ observed that urinary As levels decreased with increasing BMI in a study comparing normal weight and obese students. Scinicariello et al.^36^ reported that adults in the highest quartile of Pb levels were less likely to be obese compared to those in the lowest quartile. Heavy metals such as Pb, Cd, and Hg are highly toxic and can disrupt normal cellular metabolic functions, leading to disturbances in energy metabolism and changes in TOFAT. Thayer et al.^37^ reported that heavy metals could disrupt endocrine function, interfere with insulin signaling, and inhibit normal glucose metabolism, leading to fat breakdown. Additionally, heavy metals can cause chronic inflammation, which damages fat cells and reduces fat storage, while the inflammatory response may accelerate fat breakdown and utilization^38^. Some heavy metals bind to proteins in the body, impairing the function of fat-metabolizing enzymes^39^. Exposure to heavy metals can also cause appetite loss and digestive disorders, affecting nutrient absorption and fat storage^40,41^. To minimize the health effects of heavy metals, effective measures should be taken to reduce exposure, such as improved environmental monitoring, safer management of food and water sources, and the promotion of healthy lifestyles.

Oxidative stress, a state where there is an imbalance between the production and removal of free radicals, causes cellular damage and is linked to many metals, including Cd, Co, and Hg. These metals can lead to elevated oxidative stress levels, triggering inflammation^42,43^. For instance, Cd can replace metal ions in certain metalloenzymes, leading to enzyme inactivity and increased oxidative stress^44^. It can also deplete intracellular glutathione, an antioxidant, rendering cells unable to effectively scavenge reactive oxygen species^45^. Co has been reported to stabilize hypoxia-inducible factors, mimicking a hypoxic environment and promoting reactive oxygen generation^46,47^. Both Cd and Hg can disrupt mitochondrial function, leading to the overproduction of reactive oxygen species^48,49^. Inhaled metal particles can cause local irritation and systemic inflammation^50^. Our study found that the levels of metals were consistent with those reported in other studies, reflecting widespread metal exposure in the general U.S. population^51,52^. In the total population, we found that Cd and Co were positively related to inflammation, consistent with the finding of Zhang et al.^53^. Liu et al.^54^ also found that markers of inflammation and oxidative stress in breath were associated with occupational exposure to Co. We also found that As level was negatively correlated with SII, likely because the total As concentration was included^55^. As is an indispensable element for the human body, with evidence obtained which indicates that As is of physiological importance, especially when methionine metabolism is stressed^56^. Interestingly, we found gender differences in the metals associated with SII: As and Cd were significant in males, while Co was significant in females. Additionally, Hg was negatively correlated with SII in all populations, and more experiments are needed to elucidate the exact mechanism.

Our mediation analysis revealed that SII played a mediating role in the association between Co and TOFAT. Although Co had a direct negative effect on TOFAT, its indirect effect through SII was positive, suggesting that Co exposure increased the level of TOFAT through the mediating effect of SII. Co-induced inflammation typically involves immune cell activation, platelet aggregation, and changes in lymphocyte counts^57^. In inflammatory states, the process of lipolysis may be inhibited, leading to fat accumulation^58^. Localized adipose tissue inflammation can alter the metabolic properties of adipocytes, leading to increased fat synthesis and decreased lipolysis^59^. Our results also reflected that the mediating effect of SII between Co and TOFAT was more pronounced in females. In addition, the mediating effect of SII was not statistically different on the associations of As, Cd, and Hg with TOFAT. Further investigations are needed to elucidate the role of metals, SII, and TOFAT. The effects of metals on immune cells, adipose precursor cell differentiation, and adipogenesis can be observed in cell culture systems. Metal exposure can be simulated in experimental animals to measure SII, pro-inflammatory cytokines, insulin sensitivity, and total fat levels. Changes in SII and total fat levels and their correlation can be evaluated in high metal exposure populations. The findings of the present study suggest that inflammatory markers may play a role in the effect of metals on TOFAT levels, while the current study only explored the role of SII, a novel inflammatory marker, and therefore more inflammatory markers need to be investigated. This study had the following advantages: Firstly, the study had a large sample (3,324 participants) and a long investigation time (NHANES 2011-2018). Secondly, we first analyzed the role of SII in the relationship between metals and TOFAT. Thirdly, we included all covariates associated with TOFAT to ensure that the results of this study were stable and reliable. In addition, our study has some limitations. Firstly, some of the covariates contain reporting information bias. Secondly, this study was cross-sectional and weak in determining causal functions.

## Conclusions

In conclusion, our results indicated that most metals had a negative trend with TOFAT. In addition, Co had a positive association with TOFAT which was completely mediated by SII. Our findings have important research implications at a time when obesity health problems are becoming serious and harmful metal exposure is widespread. More investigations are needed to explore the potential biological mechanisms underlying the relationship between SII, metals, and TOFAT.

### Supplementary Information

Supplementary Information.

## Data Availability

The datasets generated and/or analysed during the current study are available in the NHANES repository, https://www.cdc.gov/nchs/nhanes/index.htm.
