# Supplementary material for: The mediation effect of Systemic Immunity Inflammation Index between urinary metals and TOFAT among adults in the NHANES dataset
Source: Sci Rep. 2024 Jun 28;14:14940. doi: 10.1038/s41598-024-65925-1 (PMC11213905; doi:10.1038/s41598-024-65925-1)
Supplement: Supplementary file 1 — Supplementary Information. [file 41598_2024_65925_MOESM1_ESM.pdf]

The mediation effect of Systemic Immunity Inflammation Index between urinary metals and TOFAT among adults in the NHANES dataset

Weipeng Zhang[1†*], Cong Zhang[2†], Dengqiu Lu[1], Junfeng Nie[1], Zhumin Hu[1], Cuiyao Xian[1], Minxing He[1]

[1]The Affiliated Panyu Central Hospital of Guangzhou Medical University, Guangzhou 511400, Guangdong, China

[2]Department of pharmacy, Guangdong Second Provincial General Hospital, Guangzhou 510317, Guangdong, China

† These authors contributed equally to this work.

*** Correspondence:**
Weipeng Zhang
zhangweipeng@pyhospital.com.cn

Table S1 Basic characteristics of the male and female.

Baseline characteristics	Male		*p*	Female		*p*
	Low	High		Low	High	
**Age (%)**			0.231			0.197
<45 year	645 (69.7)	501 (66.8)		455 (61.8)	593 (65.0)	
⩾45 year	281 (30.3)	249 (33.2)		281 (38.2)	319 (35.0)	
**BMl (%)**			0.549			<0.001
Normal	279 (30.1)	215 (28.7)		281 (38.2)	237 (26.0)	
Abnormal	647 (69.9)	535 (71.3)		455 (61.8)	675 (74.0)	
**Race(%)**			<0.001			<0.001
Mexican American	145 (15.7)	113 (15.1)		104 (14.1)	184 (20.2)	
Other Hispanic	75 (8.10)	76 (10.1)		74 (10.1)	124 (13.6)	
Non-Hispanic White	278 (30.0)	311 (41.5)		248 (33.7)	347 (38.0)	
Non-Hispanic Black	249 (26.9)	111 (14.8)		182 (24.7)	123 (13.5)	
Non-Hispanic other	179 (19.3)	139 (18.5)		128 (17.4)	134 (14.7)	
**Education level (%)**			0.436			0.804
Less than high school	177 (19.1)	154 (20.5)		121 (16.4)	161 (17.7)	
High school degree	226 (24.4)	196 (26.1)		158 (21.5)	195 (21.4)	
More than high school	523 (56.5)	400 (53.3)		457 (62.1)	556 (61.0)	
**Drinking status (%)**			0.26			0.782
Never drinker	99 (10.7)	73 (9.73)		143 (19.4)	165 (18.1)	
Ever drinker	738 (79.7)	587 (78.3)		514 (69.8)	649 (71.2)	
Current drinker	89 (9.61)	90 (12.0)		79 (10.7)	98 (10.7)	
**Smoking Status (%)**			0.003			0.36
Never smoker	546 (59.0)	392 (52.3)		510 (69.3)	622 (68.2)	
Ever smoker	200 (21.6)	214 (28.5)		123 (16.7)	175 (19.2)	
Current smoker	180 (19.4)	144 (19.2)		103 (14.0)	115 (12.6)	
**Physical activity (%)**			0.301			0.039
<100min MVPA	570 (61.6)	481 (64.1)		545 (74.0)	716 (78.5)	
⩾100 min MVPA	356 (38.4)	269 (35.9)		191 (26.0)	196 (21.5)	
**Sedentary (%)**			0.441			0.542
<360min	452 (48.8)	351 (46.8)		355 (48.2)	425 (46.6)	
⩾360min	474 (51.2)	399 (53.2)		381 (51.8)	487 (53.4)	
**Nutrient Intakes**						
**Energy (%)**			0.083			0.054
<2050 kcal	316 (34.1)	265 (35.3)		448 (60.9)	606 (66.4)	
2050-3050 kcal	362 (39.1)	256 (34.1)		225 (30.6)	245 (26.9)	
⩾3051 kcal	248 (26.8)	229 (30.5)		63 (8.56)	61 (6.69)	
**Protein (%)**			0.474			0.378
<51.5 gm	123 (13.3)	113 (15.1)		210 (28.5)	273 (29.9)	
51.5-91.5 gm	331 (35.7)	252 (33.6)		339 (46.1)	434 (47.6)	
⩾91.6 gm	472 (51.0)	385 (51.3)		187 (25.4)	205 (22.5)	
**Carbohydrate (%)**			0.241			0.127
<282 gm	466 (50.3)	397 (52.9)		543 (73.8)	711 (78.0)	
282-457.5 gm	341 (36.8)	247 (32.9)		170 (23.1)	174 (19.1)	
⩾457.6 gm	119 (12.9)	106 (14.1)		23 (3.12)	27 (2.96)	
**Fat (%)**			0.001			0.192
<45.5 gm	112 (12.1)	118 (15.7)		169 (23.0)	216 (23.7)	
45.5-101.5 gm	459 (49.6)	304 (40.5)		399 (54.2)	521 (57.1)	
⩾101.6 gm	355 (38.3)	328 (43.7)		168 (22.8)	175 (19.2)	
**SII (10^3/μL)**	304	605	<0.001	322	628	<0.001

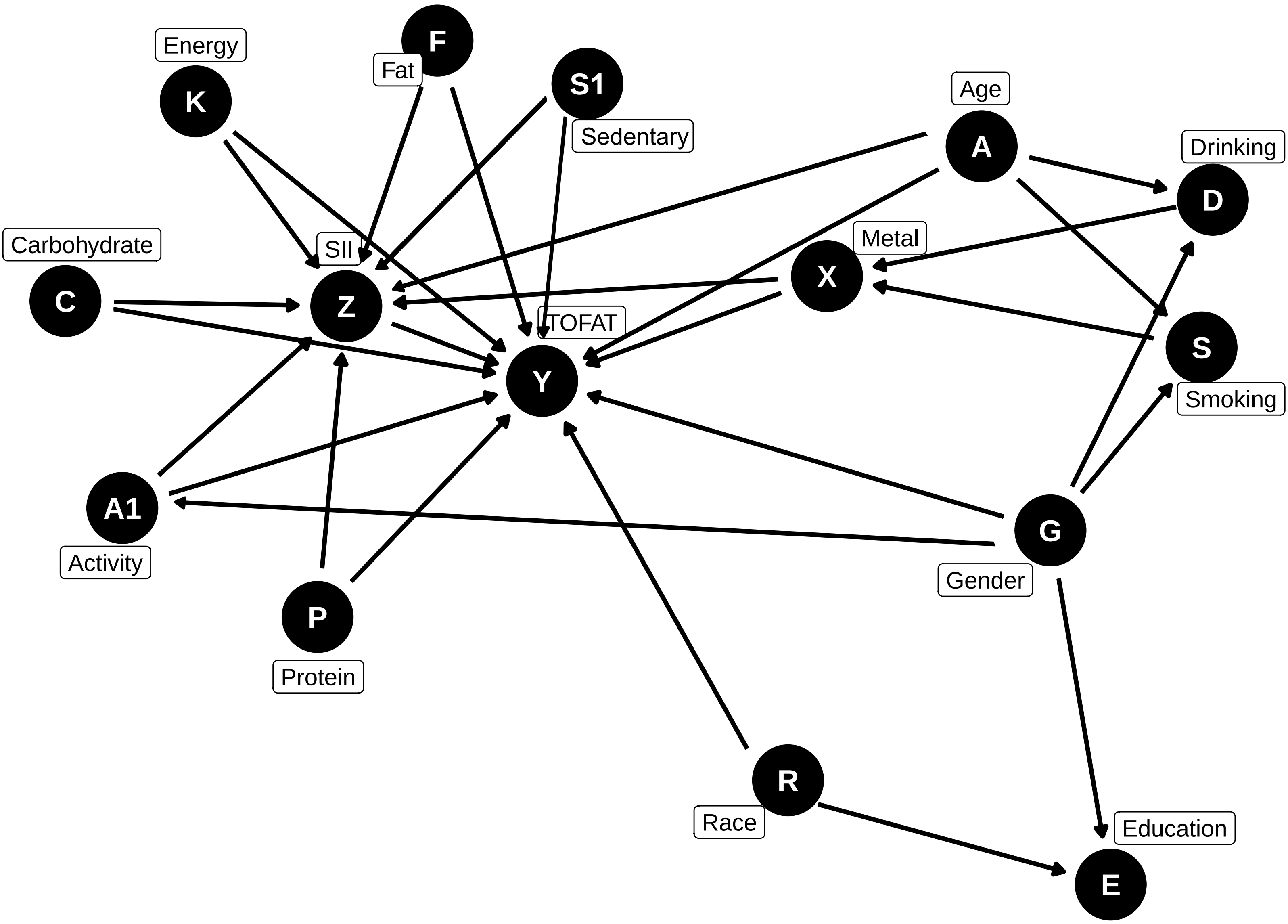

Fig.S1 Directed acyclic graphs among metals, SII, TOFAT, and all covariates.

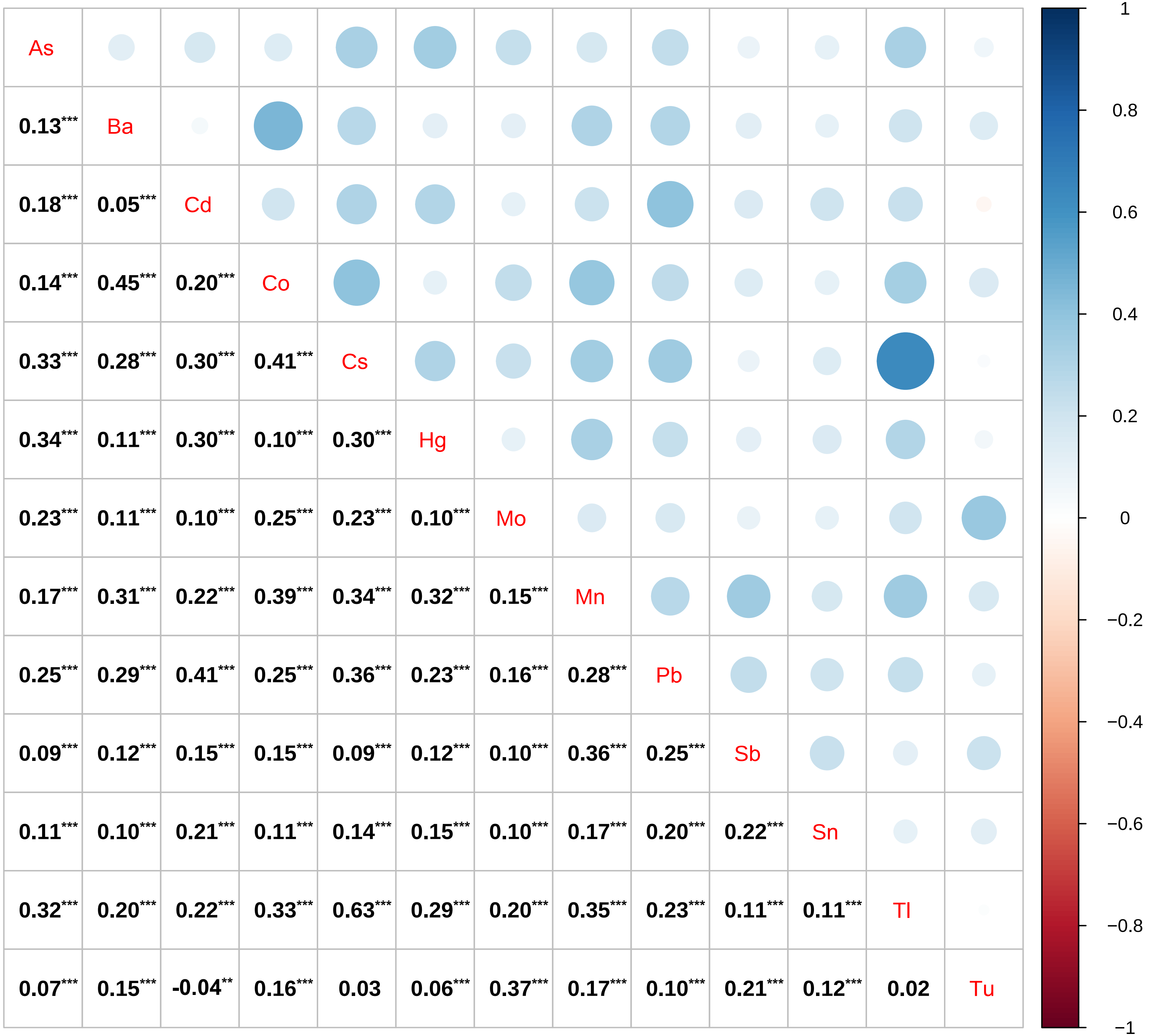

Fig.S2 Correlation analysis between the log-transform level of urinary metals. ***<0.001; **<0.05.

Table S2 The logistic regression of metals and TOFAT in NHANES.

Metals	Model	OR (95%CI)				*P* for trend
		Q1	Q2	Q3	Q4	
Ba	Model 1	Ref.	0.809 (0.664, 0.985)	0.900 (0.742, 1.091)	1.174 (0.961, 1.435)	0.057
	Model 2	Ref.	0.758 (0.618, 0.930)	0.791 (0.647, 0.967)	1.018 (0.826, 1.253)	0.732
	Model 3	Ref.	0.755 (0.615, 0.926)	0.788 (0.644, 0.963)	1.018 (0.826, 1.255)	0.725
Sn	Model 1	Ref.	1.220 (1.020, 1.459)	1.229 (1.021, 1.480)	1.669 (1.364, 2.042)	<0.001
	Model 2	Ref.	1.017 (0.844, 1.225)	0.926 (0.761, 1.126)	1.160 (0.936, 1.438)	0.428
	Model 3	Ref.	1.017 (0.843, 1.226)	0.930 (0.764, 1.131)	1.167 (0.942, 1.447)	0.394
Tu	Model 1	Ref.	0.980 (0.809, 1.186)	0.888 (0.732, 1.077)	0.970 (0.800, 1.177)	0.540
	Model 2	Ref.	0.963 (0.789, 1.174)	0.870 (0.712, 1.064)	0.936 (0.766, 1.145)	0.357
	Model 3	Ref.	0.966 (0.792, 1.178)	0.872 (0.713, 1.066)	0.941 (0.770, 1.152)	0.383

Model 1: no covariates were adjusted; Model 2: age and gender were adjusted; Model 3: age, gender, smoking, and drinking were adjusted.

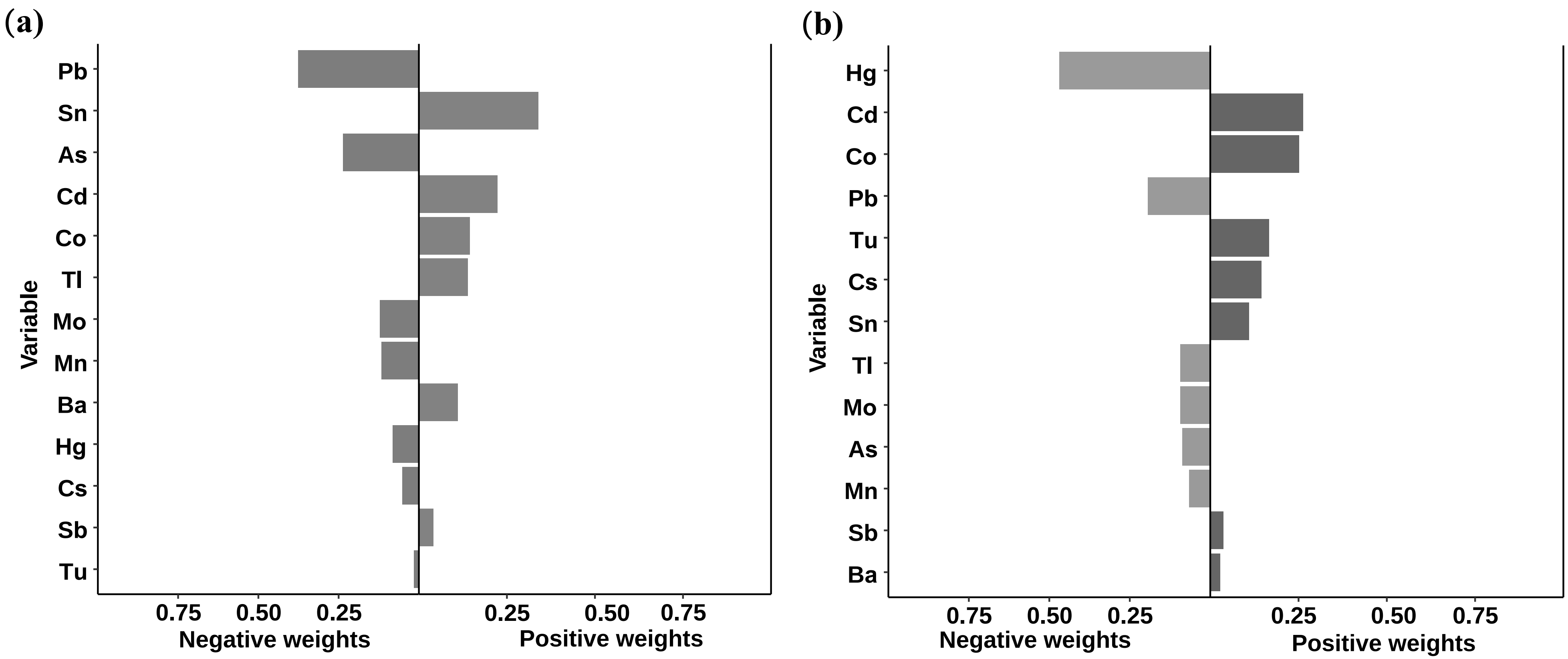

Fig.S3 The mixture effects of metals on TOFAT (a) and SII (b).

Table S3 Multivariate logistic analysis of SII and TOFAT in different groups.

Group	Model	OR (95%CI)				*P* for trend
		Q1	Q2	Q3	Q4	
Total	Model 1	Ref.	1.323 (1.091, 1.605)	1.731 (1.430, 2.097)	2.134 (1.753, 2.597)	<0.001
	Model 2	Ref.	1.253 (1.025, 1.530)	1.609 (1.320, 1.962)	1.901 (1.551, 2.330)	<0.001
	Model 3	Ref.	1.239 (1.012, 1.517)	1.629 (1.332, 1.992)	1.821 (1.481, 2.240)	<0.001
Male	Model 1	Ref.	1.292 (0.988, 1.691)	1.408 (1.074, 1.846)	1.596 (1.201, 2.119)	<0.001
	Model 2	Ref.	1.309 (0.999, 1.715)	1.418 (1.080, 1.861)	1.571 (1.181, 2.089)	0.001
	Model 3	Ref.	1.243 (0.942, 1.639)	1.423 (1.077, 1.881)	1.508 (1.124, 2.024)	0.003
Female	Model 1	Ref.	1.236 (0.925, 1.652)	1.842 (1.382, 2.456)	2.253 (1.684, 3.015)	<0.001
	Model 2	Ref.	1.201 (0.895, 1.610)	1.858 (1.389, 2.484)	2.320 (1.728, 3.114)	<0.001
	Model 3	Ref.	1.239 (0.920, 1.670)	1.918 (1.429, 2.576)	2.246 (1.667, 3.026)	<0.001

Model 1: no covariates were adjusted; Model 2: age and gender were adjusted; Model 3: age, gender, smoking, drinking, physical activity, sedentary, and nutrient intakes were adjusted.

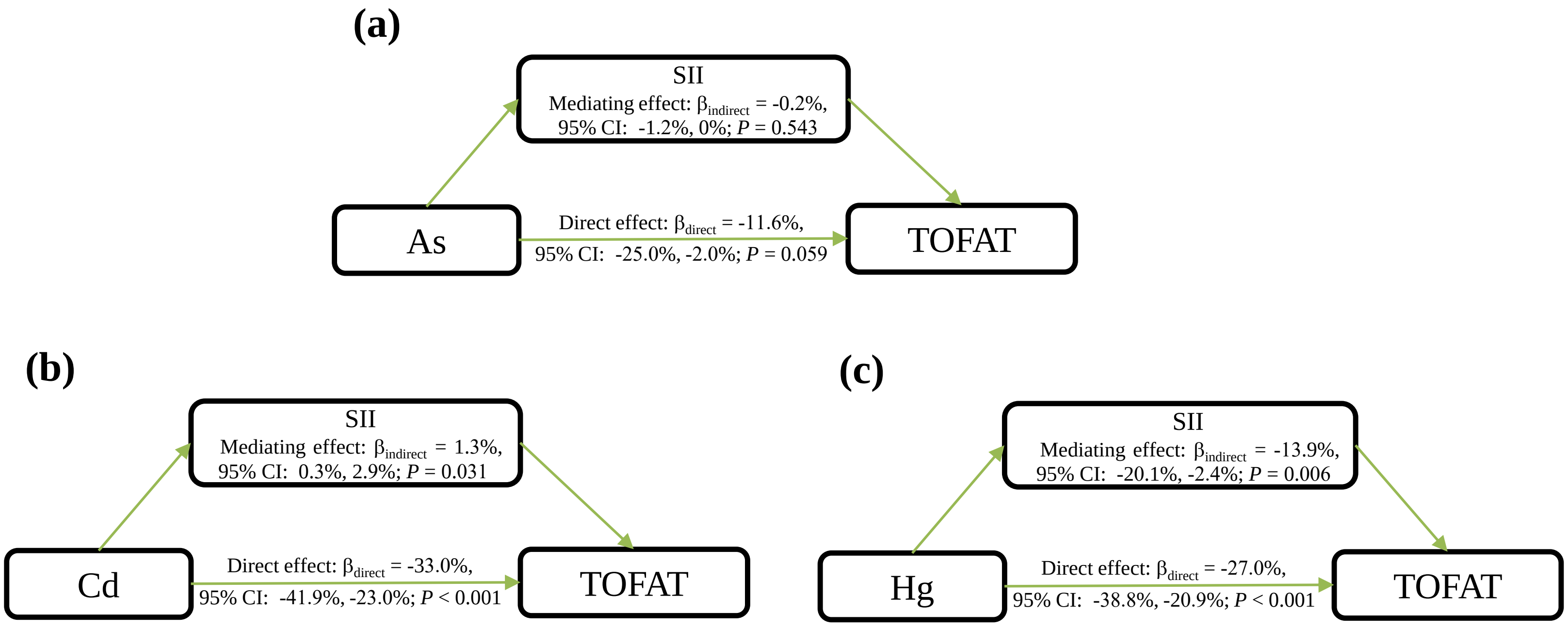

Fig.S4 Estimated proportion of the association between As, Cd, Hg, and TOFAT mediated by SII in male. Models were adjusted for age, smoking, drinking, physical activity, sedentary, and nutrient intakes.

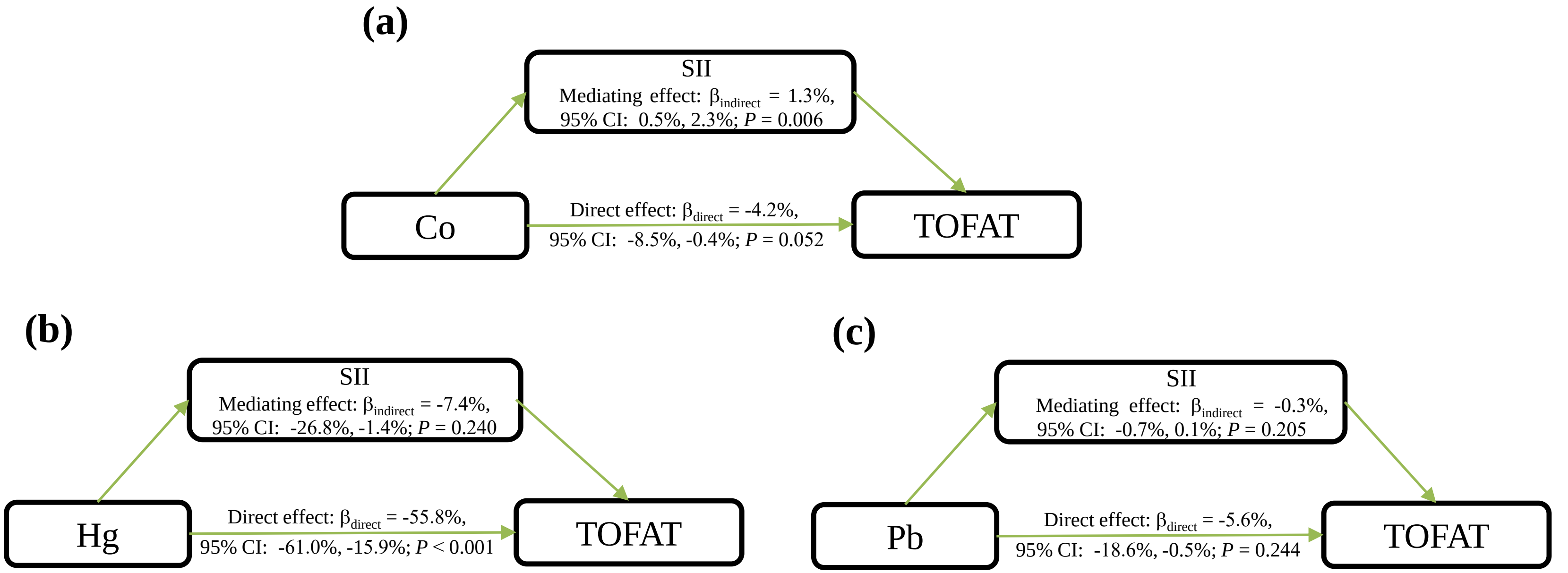

Fig.S5 Estimated proportion of the association between Co, Hg, Pb, and TOFAT mediated by SII in female. Models were adjusted for age, smoking, drinking, physical activity, sedentary, and nutrient intakes.
